# Supplementary material for: Antimicrobial Resistance: KAP of Healthcare Professionals at a Tertiary-Level Hospital in Nepal
Source: Int J Environ Res Public Health. 2021 Sep 24;18(19):10062. doi: 10.3390/ijerph181910062 (PMC8532001; doi:10.3390/ijerph181910062)
Supplement: Supplementary file 1 [file ijerph-18-10062-s001.zip › Figure S1.pdf]

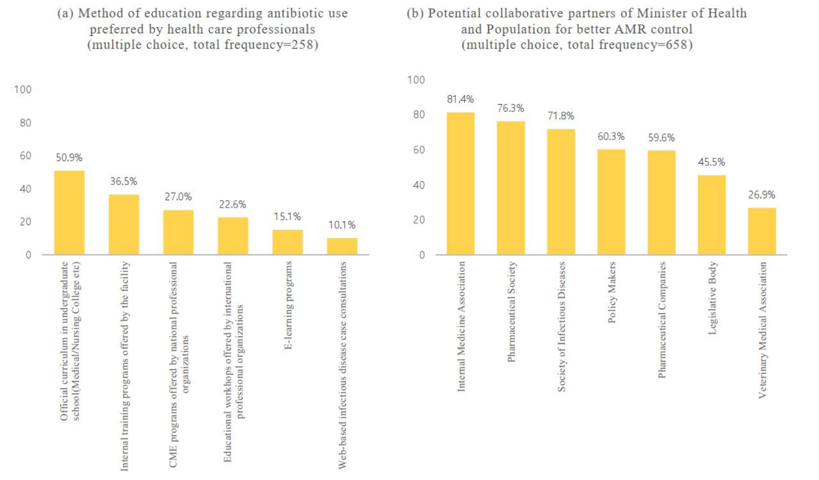
(a) Method of education regarding antibiotic use preferred by health care professionals (multiple choice, total frequency=258)
100
80
60
40
20
0
50.9%
36.5%
27.0%
22.6%
15.1%
10.1%
Official curriculum in undergraduate school(Medical/Nursing College etc)
Internal training programs offered by the facility
CME programs offered by national professional organizations
Educational workhops offered by international professional organizations
E-learning programs
Web-based infectious disease case consultations
(b) Potential collaborative partners of Minister of Health and Population for better AMR control (multiple choice, total frequency=658)
100
80
60
40
20
0
81.4%
76.3%
71.8%
60.3%
59.6%
45.5%
26.9%
Internal Medicine Association
Pharmaceutical Society
Society of Infectious Diseases
Policy Makers
Pharmaceutical Companies
Legislative Body
Veterinary Medical Association
