# Supplementary material for: Antimicrobial Resistance: KAP of Healthcare Professionals at a Tertiary-Level Hospital in Nepal
Source: Int J Environ Res Public Health. 2021 Sep 24;18(19):10062. doi: 10.3390/ijerph181910062 (PMC8532001; doi:10.3390/ijerph181910062)
Supplement: Supplementary file 1 [file ijerph-18-10062-s001.zip › suppl Document 1.pdf]

Position		Age		Department		Experience	______ years	Sex	
								M	F

Knowledge	
1. Antimicrobial Resistance means that bacteria develop the ability to defeat the antibiotics designed to kill them.	**(1) Yes / (2) No**
2. Patients with common cold symptoms need antibiotic treatment.	**(1) Yes / (2) No**
3. Antibiotics cure viral infections.	**(1) Yes / (2) No**
4. Combination of antibiotics can help prevent antimicrobial resistance.	**(1) Yes / (2) No**
5. Familiarity<br>**(1=I've never heard of it./ 2=I've heard the term but I'm not sure what it is./ 3=I've heard the term and I can explain what it is./ 4=I've used the term before./ 5=I am engaged in everyday practice)**<br>5-1. Antibiotics Stewardship Program(ASP)<br>5-2. Defined Daily Dose (DDD)<br>5-3. Days of Therapy (DOT)<br>5-4. Antibiogram	**1 2 3 4 5**<br>**1 2 3 4 5**<br>**1 2 3 4 5**<br>**1 2 3 4 5**
6. Please estimate the average proportion of Klebsiella pneumonia resistance to Ciprofloxacin in Kathmandu, Nepal (2017-2018)?	**(1) 0-25% (2) 25-50%**<br>**(3) 50-75% (4) 75-100%**<br>**(5) don't know**
7. Please estimate the average proportion of Klebsiella pneumonia resistance to Meropenem in Kathmandu, Nepal (2017-2018)?	**(1) 0-25% (2) 25-50%**<br>**(3) 50-75% (4) 75-100%**<br>**(5) don't know**

**Attitude (1=Strongly disagree/ 2=Disagree/ 3=Neither agree nor disagree/ 4=Agree/ 5=Strongly agree)**					
8. Antimicrobial Resistance is a serious public health issue in Nepal.	**1**	**2**	**3**	**4**	**5**
9. Antimicrobial Resistance is a serious issue in my facility (hospital).	**1**	**2**	**3**	**4**	**5**
10. There is a need to establish education programs on rational use of antibiotics in my facility(hospital)	**1**	**2**	**3**	**4**	**5**
11. There is a need to establish an antibiotics policy in my facility (hospital) to achieve rational antibiotic usage.	**1**	**2**	**3**	**4**	**5**
12. Cost of an antibiotic must be considered before prescription.	**1**	**2**	**3**	**4**	**5**
13. By limiting use of antibiotics, good patient care would be impaired.	**1**	**2**	**3**	**4**	**5**
14. Antibiotics are over used in our facility	**1**	**2**	**3**	**4**	**5**
15. Which method do you prefer the most to provide education on antibiotic use for health care professionals?<br>**(1) Official Curriculum in undergraduate school (Medical/Nursing College etc)**<br>**(2) Internal training programs offered by the facility**<br>**(3) CME programs offered by national professional organizations**<br>**(4) Educational workshops offered by international professional organizations**<br>**(5) E-learning programs**<br>**(6) Web-based infectious disease case consultations**					
16. Antimicrobial Resistance (AMR) should be addressed as a multi-sectoral issue that requires the collaboration of diverse partners. Please select all relevant sectors you think that the *Minister of Health and Population* should work with to AMR control.<br>**(1) Legislative body (2) Policy Makers (3) Veterinary Medical Association (5) Pharmaceutical society**<br>**(6) Pharmaceutical Companies (7) Internal Medicine Association (8) Society of Infectious Diseases**					

**Practice (1=Strongly disagree/ 2=Disagree/ 3=Neither agree nor disagree/ 4=Agree/ 5=Strongly agree)**					
If you don't have prescriptive authority, please jump to Question.19<br>17. My prescription of antibiotics is influenced by the patients' demand for antibiotics.	**1**	**2**	**3**	**4**	**5**
18. My prescription of antibiotics is influenced by the availability of the antibiotics than by the cause if diseases.	**1**	**2**	**3**	**4**	**5**
19. Bacterial confirmation and sensitivity report must be provided before antibiotic prescription.	**1**	**2**	**3**	**4**	**5**
20. If patients' medical conditions allow, IV antibiotics will be changed to oral form within 2-3days	**1**	**2**	**3**	**4**	**5**
